# Filaggrin breakdown products determine corneocyte conformation in patients with atopic dermatitis

**DOI:** 10.1016/j.jaci.2015.04.042

**Published:** 2015-12

**Authors:** Christoph Riethmuller, Maeve A. McAleer, Sjors A. Koppes, Rawad Abdayem, Jonas Franz, Marek Haftek, Linda E. Campbell, Stephanie F. MacCallum, W.H. Irwin McLean, Alan D. Irvine, Sanja Kezic

**Affiliations:** a Serend-ip GmbH, Munster, Germany; b Department of Dermatology, Our Lady's Children's Hospital, Crumlin, Dublin, Ireland; c National Children's Research Centre, Our Lady's Children's Hospital, Crumlin, Dublin, Ireland; d Coronel Institute of Occupational Health, Academic Medical Center, Amsterdam, The Netherlands; e University of Lyon 1, EA4169 “Fundamental, clinical and therapeutic aspects of the skin barrier function”, Lyon, France; f Centre for Dermatology and Genetic Medicine, Division of Molecular Medicine, Colleges of Life Sciences and Medicine, Dentistry & Nursing, University of Dundee, Dundee, United Kingdom; g Clinical Medicine, Trinity College Dublin, Dublin, Ireland

**Keywords:** Skin barrier, transepidermal water loss, atopic dermatitis, filaggrin, corneocyte, AD, Atopic dermatitis, AFM, Atomic force microscopy, CE, Cornified envelope, DTI, Dermal Texture Index, *FLG*, Filaggrin gene, LOF, Loss of function, NMF, Natural moisturizing factor, SC, Stratum corneum, SEM, Scanning electron microscopy, TEWL, Transepidermal water loss, VP, Villus-like projection

## Abstract

**Background:**

Loss-of-function (LOF) mutations in the filaggrin gene *(FLG)* are a well-replicated risk factor for atopic dermatitis (AD) and are known to cause an epidermal barrier defect. The nature of this barrier defect is not fully understood. Patients with AD with *FLG* LOF mutations are known to have more persistent disease, more severe disease, and greater risk of food allergies and eczema herpeticum. Abnormalities in corneocyte morphology have been observed in patients with AD, including prominent villus-like projections (VP); however, these ultrastructural features have not been systematically studied in patients with AD in relation to *FLG* genotype and acute and convalescent status.

**Objective:**

We sought to quantitatively explore the relationship between *FLG* genotype, filaggrin breakdown products (natural moisturizing factor [NMF]), and corneocyte morphology in patients with AD.

**Methods:**

We studied 15 children at first presentation of AD and after 6 weeks of standard therapy. We applied atomic force microscopy to study corneocyte conformation in patients with AD stratified by *FLG* status and NMF level. By using a new quantitative methodology, the number of VPs per investigated corneocyte area was assessed and expressed as the Dermal Texture Index score. Corneocytes were also labeled with an anti-corneodesmosin antibody and visualized with scanning electron microscopy.

**Results:**

We found a strong correlation between NMF levels and Dermal Texture Index scores in both acute and convalescent states (respective *r* = −0.80 and −0.75, *P* < .001 and *P* = .002). Most, but not all, VPs showed the presence of corneodesmosin abundantly all over the cell surface in homozygous/compound heterozygous *FLG* patients and, to a lesser extent, in heterozygous and wild-type patients.

**Conclusions:**

NMF levels are highly correlated with corneocyte morphology in patients with AD. These corneocyte conformational changes shed further insight into the filaggrin-deficient phenotype and help explain the barrier defect in patients with AD with *FLG* LOF mutations.

A recent study using scanning electron microscopy (SEM) showed abnormal surface structures of corneocytes from patients with atopic dermatitis (AD) that the authors named as villus-like projections (VPs).^1^ Similar structures were described as protrusions^2^ and rough corneocytes.^3^ Bead- or nipple-like elevations have also been observed in abdominal,^4^ cheek, and plantar corneocytes,^5^ as well as in 2,4,6-trinitro-1-chlorobenzene–sensitized hairless mice.^6^ They seem to be absent in forearm healthy skin^2^ or exclusively present in the periphery of corneocytes from the inner upper arm.^5^ A villous appearance with an irregular fine nodular surface pattern has also been shown in patients with ichthyosis vulgaris and in squamous cells from patients with psoriasis.^7^ In most of these studies, VPs were observed qualitatively, and only in one study were the VPs determined semiquantitatively,^5^ suggesting a correlation between VP numbers and skin barrier function, as assessed based on transepidermal water loss (TEWL). The nature and cause of VPs on the stratum corneum (SC) surface is not well understood. Several mechanistic suggestions have been proposed for the occurrence of VPs, including disturbed organization of the cytoskeleton on desmosome disruption, immature and fragile cornified envelopes (CEs), and attachment sites of desmosomes.^1^, ^2^, ^6^, ^8^ Rankl et al^4^ showed that staining for corneodesmosin protein mostly matched the beadlike topographic features, although not all of these structures showed corneodesmosin immunoreactivity.

Because filaggrin (gene name *FLG*) is a component of the CE^9^ and filaggrin-deficient corneocytes display gene dose-dependent alterations in CE structure,^10^ we aimed to investigate the relationship between VPs on the SC with levels of filaggrin degradation products in children with AD. The filaggrin degradation products histidine, pyrrolidone-5-carboxylic acid, and urocanic acid can be used as an indirect measure of filaggrin expression that is dependent not only on *FLG* loss-of-function (LOF) mutations but also on other factors, including genetic factors, filaggrin degradation pathway factors,^11^ and both local and systemic inflammation.^12^, ^13^ Furthermore, because these products are the main source of the constituents of natural moisturizing factor (NMF) and contribute to SC hydration, their levels might influence structural conformation of the CE.

In this study we used high-resolution atomic force microscopy (AFM) to investigate the topography of corneocytes in patients with AD in relation to *FLG* genotype and levels of filaggrin degradation products. AFM provided nanoscale 3-dimensional resolution of native corneocytes collected by means of adhesive tape stripping. AFM involves a sharp tip at the end of a soft silicon cantilever touching and scanning the surface of a sample. Because of the change in topography, the deflection of the cantilever is transformed into a 3-dimensional image. Recently, we have developed and evaluated a software method through which VP surfaces on the corneocyte can be quantitatively determined (the Dermal Texture Index [DTI]; technical manuscript in preparation, full details available on request from the authors [CR]). We measured DTI scores in corneocytes of children with active AD at first presentation and after 6 weeks of standard topical therapy with skin care regimens and appropriate topical steroids. In addition to DTI scores, we measured NMF levels; skin barrier function, as assessed based on TEWL; and severity of AD based on the SCORAD score.^14^ Next, we investigated the distribution of corneodesmosome remnants by using SEM and corneodesmosin immunocytochemical labeling.

## Methods

### Study population

Patients with AD were recruited from a dedicated AD clinic in a tertiary referral center. An experienced pediatric dermatologist (ADI, MAM, or both) made the diagnosis and recorded the disease phenotype. All patients met the United Kingdom diagnostic criteria^15^ and had moderate or severe disease. Exclusion criteria from the study included patients who had pyrexial illness in the preceding 2 weeks; those who had received immunosuppressive systemic therapy, such as oral corticosteroids, in the preceding 3 months; and those whose ancestry was not exclusively Irish (4/4 grandparents). The study was conducted in accordance with the Helsinki Declarations and was approved by the Research Ethics Committee of Our Lady's Children's Hospital, Dublin, Ireland. Full written informed consent was obtained from all patients' parents. The children were treatment naive at presentation and were assessed at first presentation and after 6 weeks of standard treatment with skin care regimens and appropriate topical steroids.

### Severity assessment

The severity of a patient's AD was assessed by using the SCORAD index. A single dermatologist performed all SCORAD measurements. SCORAD is one of the most valid and reliable instruments to assess the clinical severity of AD.^16^ SCORAD is a composite score on a scale of 0 to 103 that incorporates both objective physicians' estimates of extent and severity and subjective patient or parental assessments of itch and sleep loss.^17^ SCORAD is internally consistent, responsive, and interpretable and has adequate interobserver reliability (Cohen κ = 0.82, *P* < .001).^18^

### Biophysical measurements of the SC

All topical therapies, including emollients, were withheld from the patients' upper limbs for 48 hours before skin biophysical measurements were performed. All measurements were done in standardized environmental conditions (room temperature, 22°C to 25°C; humidity levels, 30% to 35%). Before testing, the patient's forearm was acclimatized to this controlled environment for a minimum of 10 minutes. All measurements were done by the same investigator and on a clinically unaffected area of skin on the volar forearm. TEWL was determined by using a Tewameter 300 (Courage and Khazaka Electronic GmbH, Cologne, Germany).

### Sampling of the SC by using tape stripping

The SC was sampled by using the previously described method.^19^ A clinically unaffected site on the patient's volar forearm, where the TEWL measurement was also taken, was used for SC sampling. Circular adhesive tape strips (3.8 cm^2^, D-Squame; Monaderm, Monaco, France) were attached to volar forearm skin and pressed for 10 seconds with a constant pressure (225 g/cm^2^) by using a D-Squame Pressure Instrument D500 (CuDerm, Dallas, Tex). The tape strip was then gently removed and placed in a closed vial. Eight consecutive tape strips were sampled, all from the same site. The tape strips were immediately stored at −80°C until analysis.

### *FLG* genotyping

All patients were screened for the 9 most common *FLG* mutations found in the Irish population (R501X, Y2092X, 2282del4, R2447X, S3247X, R3419X, 3702X, S1040X, and G1139X) from DNA extracted from a blood sample. The methods used have been previously described.^20^

### NMF determination

NMF analysis was performed on the fifth consecutive strip, according to methods described in detail elsewhere.^19^ Briefly, each tape strip was extracted with 25% (wt/wt) ammonia solution. After evaporation of the ammonia extract, the residue was dissolved in 250 μL of pure water and analyzed by using HPLC. The NMF concentration was normalized for the protein amount determined with a Pierce Micro BCA protein assay kit (Thermo Fischer Scientific, Rockford, Ill; referred to as the Pierce assay) to compensate for a variable amount of the SC on the tape.

### Skin nanotexture analysis (DTI)

Corneocytes from patients were analyzed with AFM, as previously described.^21^ Briefly, in each case the seventh tape strip was subjected to AFM measurements carried out with a Multimode AFM equipped with the Nanoscope III controller and software version 5.30sr3 (Digital Instruments, Santa Barbara, Calif). Silicon-nitride tips on V-shaped gold-coated cantilevers were used (0.01 N/m, MLCT; VEECO, Mannheim, Germany). Imaging was performed at ambient temperature with forces of less than 1 nN and 1 to 3 scan lines per second (1-3 Hz) with 512 × 512 pixel resolution. For texture analysis, subcellular scan areas of 20 μm^2^ were recorded. Ten random images were analyzed from each sample.

Topographic cell-surface data were analyzed with the nAnostic method, applying custom-built proprietary algorithms (Serend-ip GmbH, Munster, Germany). The principle of this method has been described elsewhere.^22^ Briefly, each nanostructure protruding from the mean surface level was morphometrically evaluated. These objects were then filtered by size and shape through computer vision. At this stage, only structures of positive local deviational volume smaller than 500 nm in height and with an area of less than 1 μm^2^ were considered. The DTI score is the count of identified objects per image (a mean value from 10 randomly recorded images).

### Corneodesmosin immunolabeling

Corneocytes from 3 patients with different *FLG* mutation genotypes collected on D-squame discs were labeled with an anti-corneodesmosin antibody and visualized with SEM, as described elsewhere.^23^ Briefly, the native cells exposed to the mouse mAb to corneodesmosin (diluted 1:100; Abnova, Jhongli City, Taiwan) were immunogold labeled with the goat anti-mouse Ultra Small probe (0.8 nm, diluted 1:10; Aurion, Wageningen, The Netherlands). The labeling was silver enhanced with the BBI kit (BBI Solutions, Cardiff, United Kingdom), and the samples, after dehydration in ethanol, were observed in a partial vacuum by using secondary and backscattered electron detection modes.

### Statistics

Data were checked for normality by using the Shapiro-Wilk test. The relationship between DTI scores and clinical parameters was tested either by using the Pearson correlation test or Spearman rank correlation if the variables were not normally distributed or the relationship between the variables was not linear. Because of skewed distribution, DTI scores and NMF levels were log-transformed before linear regression analysis. Differences in the investigated parameters (DTI score, TEWL, SCORAD score, and NMF level) between 2 measurement points (0 and 6 weeks) were tested by using the paired 2-sided *t* test (NMF and DTI score) or by using the Wilcoxon matched signed-rank test in the case of deviation from normal distribution (TEWL and SCORAD score). Differences in DTI scores among 3 *FLG* genotypes were tested by using the Kruskal-Wallis test, followed by Dunn multiple comparison. The relationship between the DTI score as a dependent variable versus the SCORAD score and NMF level as dependent variables was tested by using a linear regression model with SPSS software (version 22; IBM, Somers, NY). For other statistical analyses, GraphPad Prism version 5.00 software for Windows (GraphPad Software, San Diego, Calif) was used. A *P* value of less than .05 was considered statistically significant.

## Results

**Fig 1:**
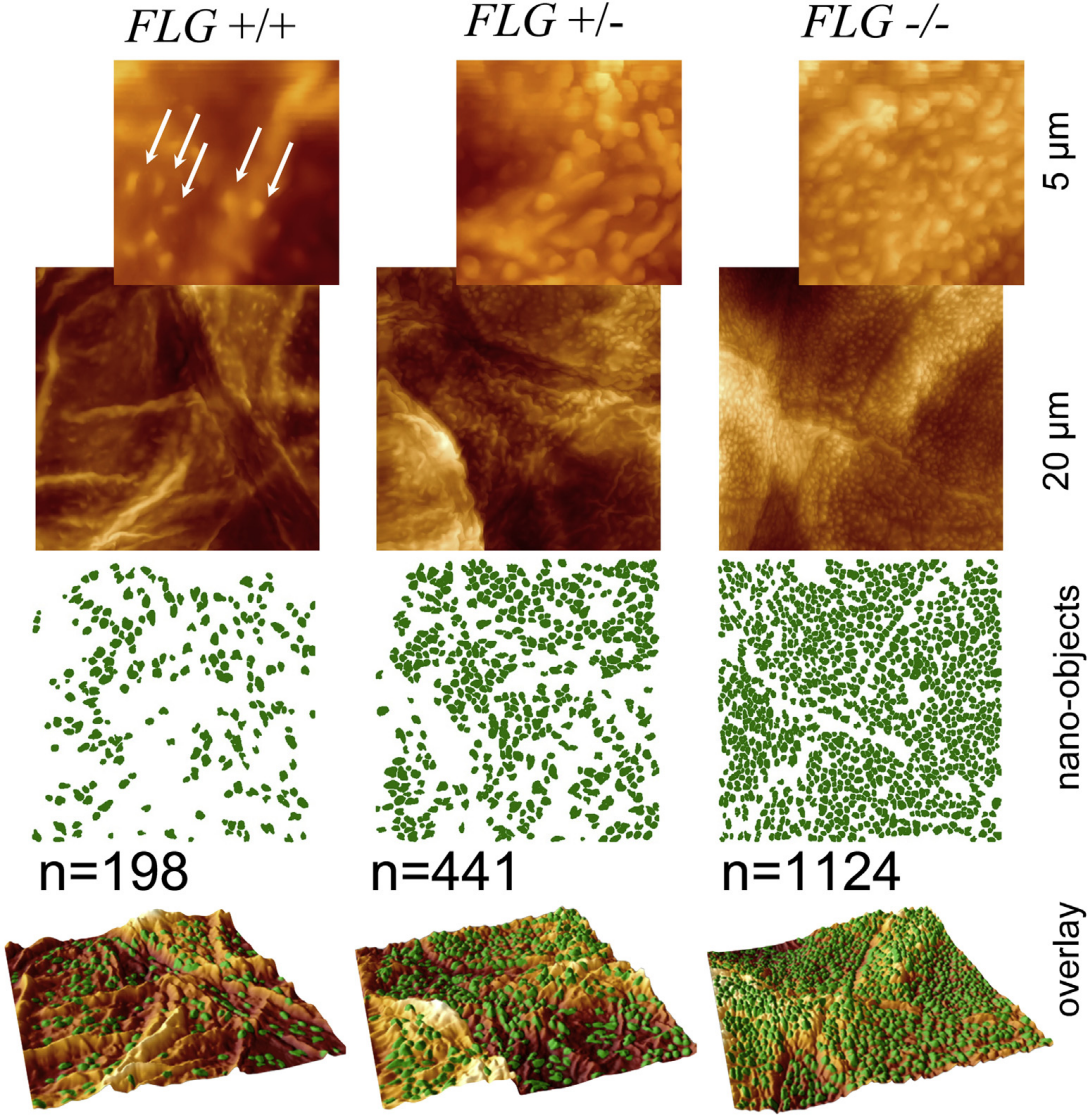
Representative AFM images of the surfaces of corneocytes sampled from patients with AD with 3 different *FLG* mutation genotypes: +/+, wild-type homozygote; +/−, heterozygote for *FLG* LOF mutation; −/−, compound heterozygote or homozygote for *FLG* LOF mutation. On simple inspection, numbers of VPs were clearly increased in carriers of *FLG* mutations.

**Fig 2:**
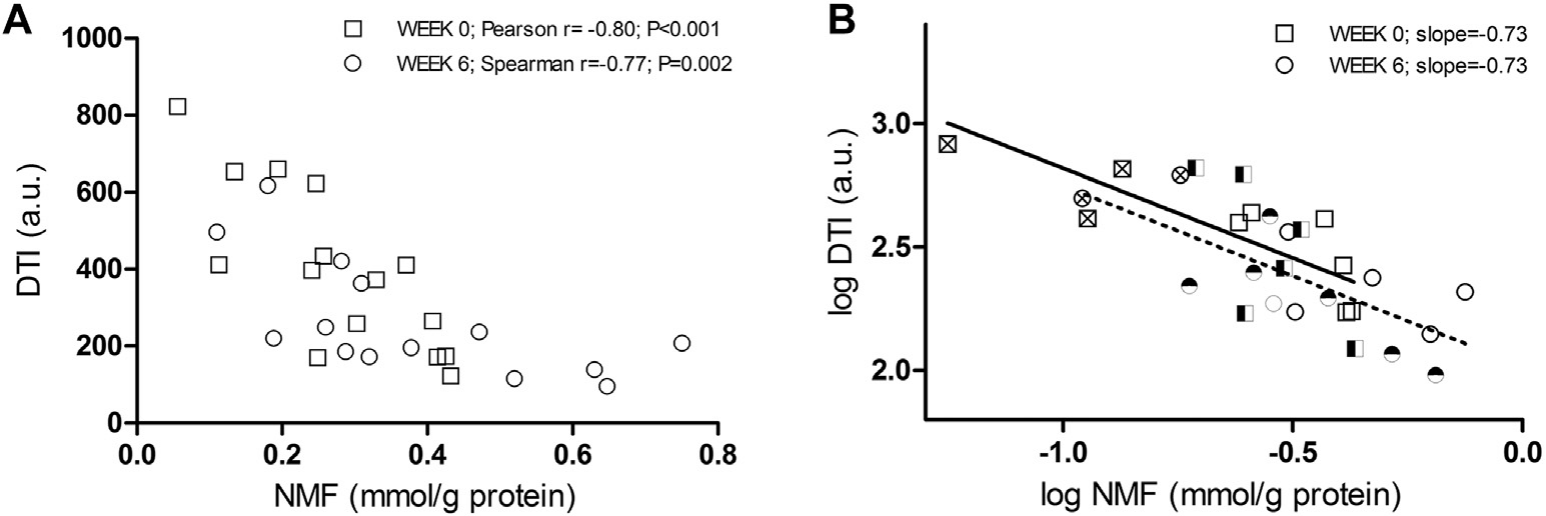
**A,** Relationship between DTI scores and NMF levels at first presentation of disease *(squares)* and after 6 weeks of topical therapy with skin care regimens and appropriate topical steroids *(circles)*, with corresponding correlation coefficients *(r)*. **B,** Linear regression analysis of log-transformed DTI scores and NMF levels at first presentation of disease *(squares)* and after 6 weeks of topical therapy with skin care regimens and appropriate topical steroids *(circles)*. □ ○, Patients with AD wild-type with respect to *FLG* LOF mutations; ◓, ◨, patients with AD heterozygous for *FLG* LOF mutations; ⊗⊠, patients with AD homozygous or compound heterozygous for *FLG* LOF mutations.

Demographic characteristics of the investigated populations and values of measured parameters are presented in Table I. Fig 1 shows representative AFM images of the surfaces of corneocytes sampled from patients with AD with 3 different *FLG* mutation genotypes. On simple inspection, VP numbers were clearly increased in carriers of *FLG* mutations. The DTI score, which quantifies the number of VPs per investigated surface area, showed a trend toward higher mean values in the carriers of *FLG* mutations compared with *FLG* wild-type subjects at week 0 (427.0 and 336.2, respectively) and after 6 weeks of therapy (296.6 and 224.3, respectively), although the differences did not reach statistical significance (data not shown). At week 6, however, the DTI in the *FLG*^−/−^ group was significantly higher than in the *FLG*^+/+^ group (respective median values were 496.8 and 208.2, respectively; *P* < .05, as assessed by using the 2-tailed Mann-Whitney test), whereas there was no significant difference in SCORAD scores between these 2 *FLG* genotype groups. When DTI scores were plotted against the NMF levels, a significant correlation was observed at both weeks 0 and 6 (respective correlation coefficients amounted to −0.80 and −0.75, *P* < .001 and *P* = .002, respectively; Fig 2, *A*). VP numbers reach a plateau at normal NMF levels (approximately 0.5 mmol/g protein).

**Fig E1:**
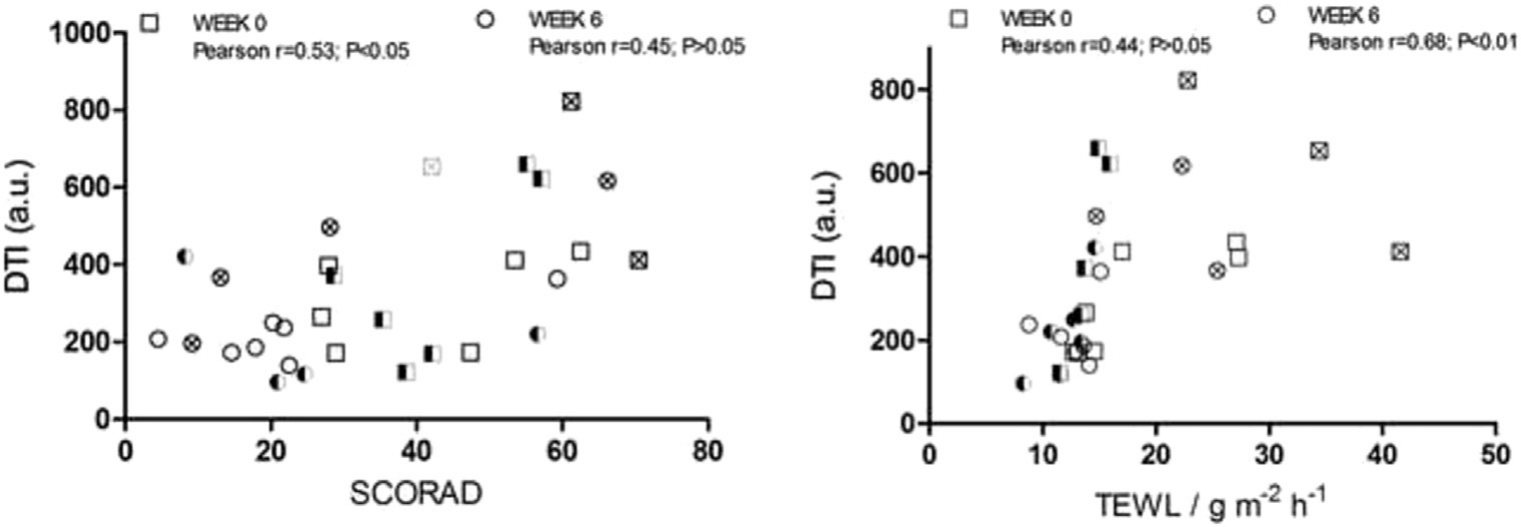
Relationship between DTI scores and clinical parameters at first presentation of disease *(squares)* and after 6 weeks of topical therapy with skin care regimens and appropriate topical steroids *(circles)*. □ ○, Patients with AD with respect to *FLG* LOF mutations; ◓, ◨, patients with AD heterozygous for *FLG* LOF mutation; ⊗⊠, patients with AD homozygous or compound heterozygous for *FLG* LOF mutations.

Regression analysis of log-transformed values of DTI scores and NMF levels showed almost identical regression coefficients for 0 and 6 weeks (−0.726 and −0.730, respectively; Fig 2, *B*). The relationship of DTI scores with TEWL and SCORAD scores (see Fig E1 in this article's Online Repository at www.jacionline.org) was weaker than that of DTI scores and NMF levels. Furthermore, in a linear regression model with the DTI score as a dependent variable versus the NMF level and SCORAD score, only NMF levels showed a significant effect on DTI scores (*P* = .005 and .015, respectively, for weeks 0 and 6; see Table E1 in this article's Online Repository at www.jacionline.org).

**Fig 3:**
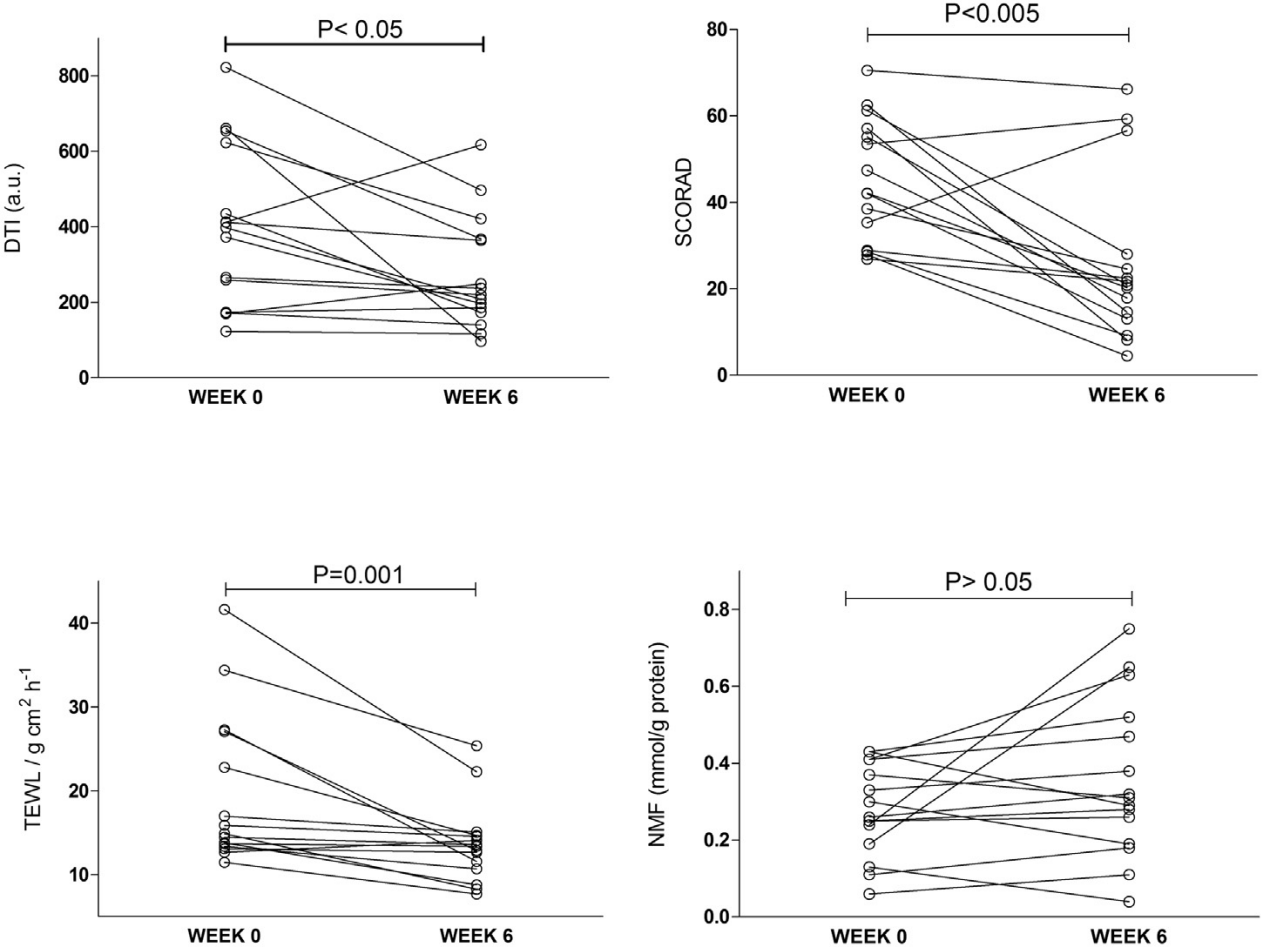
TEWL, SCORAD score, DTI score, and NMF level at first presentation of disease and after 6 weeks of topical therapy with skin care regimens and appropriate topical steroids.

Changes in DTI scores, NMF levels, TEWL, and SCORAD scores measured at the first presentation of disease and after 6 weeks of standard topical therapy with skin care regimens and appropriate topical steroids are shown in Table I and Fig 3. Although the skin barrier, as measured based on TEWL and SCORAD scores, significantly improved after 6 weeks of therapy, NMF levels and DTI scores did not mirror these improvements in all patients.

**Fig 4:**
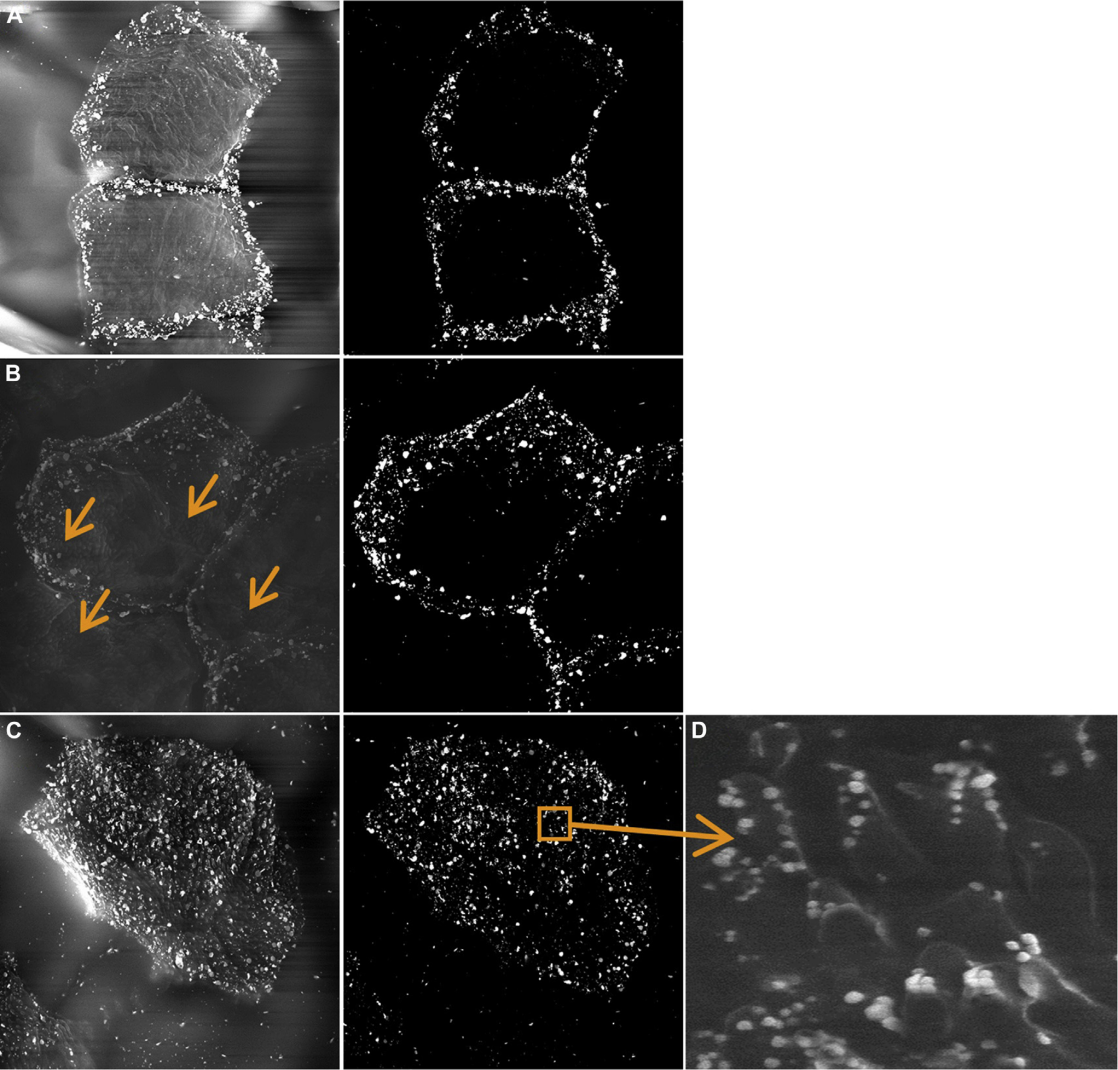
SEM immunolabeling of corneodesmosin. **A,** Corneodesmosomes at the cell surface of a patient wild-type with respect to *FLG* LOF mutations (*FLG*^+/+^). **B,** A patient heterozygous for *FLG* LOF mutations (*FLG*^+/−^). **C,** A patient homozygous for *FLG* LOF mutations (*FLG*^−/−^). Insert in Fig 4, *C*, Corneodesmosin-expressing junctions present at the tops of the VPs in the patient homozygous for *FLG* LOF mutations. *Arrows* in Fig 4, *B*, show the presence of VPs (not labeled for corneodesmosin).

Representative SEM images of D-Squames after immunogold labeling are shown in Fig 4 for 3 patients with AD of different *FLG* genotype status. The high abundance of VPs on the corneocytes obtained from an *FLG*^−/−^ subject (Fig 4, *C*) was confirmed by means of SEM. The VPs were decorated at their tips with corneodesmosin labeling, indicating the presence of disrupted corneodesmosome structures (Fig 4, *D*). The corneocytes of a homozygous subject (*FLG*^−/−^) demonstrated labeling over the entire surface (Fig 4, *C*). In contrast, in a patient who is wild-type with respect to *FLG* mutations (*FLG*^+/+^; Fig 4, *A*), the labeling was almost exclusively distributed on the lateral rims of the cell. In the heterozygous patient (*FLG*^+/−^; Fig 4, *B*) the central area of corneocytes remained largely free of the label, even though it was partially occupied by the VPs (Fig 4, *B*, arrows).

## Discussion

Filaggrin deficiency results in a definite skin barrier defect, but the pathomechanisms underlying this defect are poorly understood.^11^ Within the corneocytes, filaggrin aggregates intermediate keratin filaments that are linked to the corneodesmosomes, which interconnect the corneocytes, providing a physical barrier structure at the top of the skin.^24^, ^25^ Together with keratin filaments, filaggrin has been proposed to provide a scaffold for the assembly of structural proteins, such as involucrin, loricrin, and small proline-rich proteins, which are cross-linked by several transglutaminases to form the CE.^24^, ^25^ Some CE proteins serve as an anchor for attachment of ceramides, and thus lack of filaggrin might also affect the structural organization of the intercellular SC lipid lamellae responsible for barrier function.

In the present study we demonstrate that deficiency of filaggrin is associated with altered topography of the corneocyte surface, likely caused by defects in the CE. In a recent study^2^ similar villous structures were observed on the palmar skin of healthy subjects, although not on forearm skin, which is in contrast to the present study. We found that levels of filaggrin degradation products (NMF) used as a marker of filaggrin expression^12^, ^26^ were strongly associated with corneocyte VP numbers. These corneocytes were sourced from the upper middle part of the SC (seventh strip); however, the same pattern concerning distribution of VPs was also seen in the more superficial strips (eg, strip number 4; data not shown). VP numbers were more closely related to NMF levels than to SCORAD scores, suggesting that the absence of filaggrin is important for the formation of VPs rather than inflammation *per se*. This is supported by similar regression coefficients of the DTI score versus NMF level relationship at weeks 0 and 6, despite the sharp decrease in SCORAD scores. Furthermore, in a linear regression model with the DTI score as a dependent variable versus the NMF level and SCORAD score as independent variables, only NMF levels showed a significant effect on DTI scores at both weeks 0 and 6. Local inflammation might have affected the presence of DTI scores indirectly by influencing NMF levels, an effect that previously has been shown *in vitro* and *in vivo*.^12^, ^13^ This might explain the lack of a significant difference in DTI scores between patients with AD with *FLG* LOF mutations and patients with AD without *FLG* LOF mutations, although the former group tended to have higher DTI scores, and the lack of statistical significance seen here might simply be due to a lack of power in this study. Furthermore, at week 6, the *FLG*^−/−^ patients, in whom inflammation is controlled and NMF levels are mainly influenced by *FLG* LOF mutations, had significantly higher DTI scores compared with *FLG*^+/+^ patients, despite clinical improvement, as measured based on SCORAD scores. Also of note is our observation that the relationship between TEWL and DTI scores was significant at 6 weeks (after anti-inflammatory therapy) but not at week 0 (see Fig E1). This suggests a relationship between corneocyte conformation as measured by DTI scores and barrier function (TEWL).

During the transition from the stratum compactum to the stratum disjunctum, corneocyte morphology and mechanical properties change from a “fragile” and soft to a more robust, smooth, and “rigid” phenotype.^27^, ^28^, ^29^ This transition process is accompanied by loss of nonperipheral corneodesmosomes because only peripheral corneodesmosome attachments connecting consecutive layers of corneocytes remain.^28^, ^29^ Interestingly, we observed corneodesmosin on the tips of VPs, all over the cell surface in *FLG*^−/−^ patients, and, to lesser extent, in heterozygous patients, which suggests changes in their maturation because of a disturbed terminal differentiation program. As discussed by Rawlings^28^ and shown by Watkinson and Rawlings,^30^ the loss of nonperipheral corneodesmosomes and CE maturation changes seem to parallel filaggrin degradation. Lack of filaggrin in the CE and between the keratin filaments might lead to conformational changes, and the adhesive portions of the peripheral corneodesmosomes might become less accessible for degradation enzymes. In addition to the direct effect of filaggrin, the existence of VPs might also be caused by a reduction in filaggrin degradation products and reduced hydration of the SC. Matsumoto et al^6^ observed the emergence of villi on the rear surfaces of corneocytes after topical exposure to the contact allergen 2,4,6-trinitrochlorobenzene, which caused dry and inflamed skin. However, the villi disappeared after topical treatment with a moisturizer at a higher rate than after topical corticosteroid therapy. The surfactant-induced xerosis led to a considerable increase of the immature and fragile CE phenotype.^29^ The perturbation of CE maturation coincided with reduced hydrolysis of corneodesmosomes, which was paralleled by altered activity of transglutaminase. Recently, we have shown that exposure to sodium lauryl sulfate caused a dramatic decrease in NMF levels in the SC,^31^ and therefore the changes in corneocyte maturation might have also been caused by the lack of NMF. Interestingly, also in the study of Harding et al,^29^ the balance between the 2 CE phenotypes was recovered after treatment with a moisturizer, emphasizing the importance of SC hydration for the maturation process.

The size of the VPs (ie, several hundreds of nanometers: average height, 350 nm; width at half-maximum, 250-400 nm) and their high abundance is intriguing. The CE is approximately 20 nm thick, implying that considerable mechanical force lies behind its protrusion. The present results do not allow firm conclusions to be drawn regarding the relationship between VPs and retention of the nonperipheral corneodesmosomes because the presence of VPs was not always accompanied by central distribution of corneodesmosin. The persistence of VPs in both the acute and convalescent phases of AD with *FLG* loss-of-function mutations offers an intriguing insight into the persistent abnormalities in “normal” or “unaffected” AD skin, an area of great interest.^32^ The persistence of an underlying physical and structural abnormality, even in light of apparent clinical improvement, might explain why patients with AD with *FLG* loss-of-function mutations have more severe and persistent disease,^33^ why they are more likely to have eczema herpeticum,^34^ and why they have more food allergies.^35^

In conclusion, we have shown for the first time a significant structural difference in corneocytes in patients with AD with *FLG* loss-of-function mutations that can be quantitatively measured. These structural changes correlate well with NMF levels and persist despite apparent clinical improvement and might explain some of the observed phenotypic differences in patients with AD with *FLG* loss-of-function mutations.

**Clinical implications**

Corneocytes of patients with AD with *FLG* loss-of-function mutations are morphologically distinct both in active disease and in convalescence from those of patients with AD without *FLG* LOF mutations. These structural differences can explain clinical differences between AD endophenotypes and facilitate assessment of therapeutic interventions.

## Figures and Tables

**Table E1:** Linear regression model for DTI score as the dependent variable and NMF level and SCORAD score as predicting variables at baseline (treatment naive) and after 6 weeks of treatment

Coefficients∗						
Model		Unstandardized coefficients		Standardized coefficients	*t*	Significance (*P* value)
B	SE	β
Baseline∗						
1	(constant)	680.806	222.435		3.061	.010
	NMF0	−1292.165	375.588	−0.729	−3.440	.005
	SCORAD0	1.664	3.159	0.112	0.527	.608
After 6 wk of treatment†						
1	(constant)	470.605	78.408		6.773	.000
	NMF6	−514.723	178.133	−0.654	−3.012	.015
	SCORAD6	−0.200	0.560	−0.081	−0.314	.727

*DTI0*, DTI score at o weeks; *DTI6*, DTI score at 6 weeks; *NMF0*, NMF level at 0 weeks; *NMF6*, NMF level at 6 weeks; *SCORAD0*, SCORAD score at 0 weeks; *SCORAD6*, SCORAD score at 6 weeks.

∗ The dependent variable is DTI0, and predictors (constant) are SCORAD0 and NMF0.

† The dependent variable is DTI6, and predictors (constant) are SCORAD6 and NMF6.

**Table I:** Demographic data of patients, DTI scores, and NMF levels assessed at baseline (treatment naive) and after 6 weeks of treatment

Sex	Age (mo)	No. of *FLG* mutations	SCORAD score		TEWL (g/m^2^ h)		DTI score (AU)		NMF (mmol/g)	
Week 0	Week 6	Week 0	Week 6	Week 0	Week 6	Week 0	Week 6
M	9.3	0	28.9	22.5	12.7	14.1	172.0	139.8	0.41	0.63
M	5.3	0	62.5	14.6	27.1	12.9	434.4	172.7	0.26	0.32
M	5	0	27.9	4.5	27.3	11.6	397.5	208.2	0.24	0.75
M	9	0	53.5	59.3	17	15.1	411.2	363.5	0.37	0.31
M	9.5	0	26.9	21.8	13.8	8.8	265.8	237.3	0.41	0.47
M	8.5	0	47.4	17.9	14.5	13.6	173.4	185.7	0.43	0.29
M	2.3	1	42.1	20.3	13.1	12.7	170.1	249.3	0.25	0.26
F	57.3	1	38.5	24.6	11.5	7.7	122.5	116.3	0.43	0.52
M	10	1	55.1	20.9	14.9	8.3	660.6	96.3	0.19	0.65
F	6	1	57.1	8.2	15.9	14.6	623.0	421.0	0.25	0.28
M	6.25	1	35.3	56.6	13.4	10.7	259.1	219.9	0.30	0.19
M	8.25	1	28.6	9.2	13.7	13.4	372.8	196.3	0.33	0.38
M	6.8	2	70.5	66.2	41.6	22.3	412.4	617.3	0.11	0.18
M	28	2	61.2	28.1	22.8	14.7	822.5	496.8	0.06	0.11
M	5.75	2	42	13.1	34.4	25.4	653.8	367.2	0.13	1∗

*F*, Female; *M*, male.

∗ Analysis failed.
